# Toxicity effects of cadmium and β-cypermethrin on zebrafish by single and combined exposure: oxidative stress and histopathological evaluation

**DOI:** 10.3389/ftox.2025.1674140

**Published:** 2025-11-17

**Authors:** Xinkun Fu, Suxian Yang, Yi Zhang, Kai Luo, Yonglin Sun, Yuqi Li

**Affiliations:** 1 School of Food Science and Chemical Engineering, Hubei University of Arts and Science, Xiangyang, China; 2 Analysis and Testing Center, School of Food Science and Chemical Engineering, Hubei University of Arts and Science, Xiangyang, China

**Keywords:** zebrafish, cadmium, β-cypermethrin, enzyme activity, toxicity effect

## Abstract

**Introduction:**

This study investigated the effects of cadmium (Cd) and β-cypermethrin (β-CP), both singly and in combination, on oxidative stress responses and tissue morphology in zebrafish.

**Methods:**

Through acute exposure experiments, we evaluated the acute toxicity and behavioral responses of zebrafish to these two compounds.

**Results:**

The LC50 values of contaminants for zebrafish at 24 h, 48 h, 72 h, and 96 h were 25.36, 22.94, 20.36, and 17.83 mg/L for Cd, and 6.41, 4.96, 4.23, and 3.75 μg/L for β-CP, respectively. The results showed that β- CP exhibited higher acute toxicity compared to Cd, with pronounced toxic reactions including body curling, convulsions, accelerated operculum flapping, and significantly reduced swimming ability. In contrast, cadmium (Cd) elicited milder acute toxicity symptoms yet significantly disrupted key oxidative stress parameters, including superoxide dismutase (SOD), catalase (CAT), and malondialdehyde (MDA). During the chronic co-exposure phase, the combined treatment of Cd and β-cypermethrin (β-CP) resulted in more severe toxicity than individual exposures, as supported by marked bioaccumulation of both contaminants and extensive histopathological impairments—such as neuronal degeneration in the brain, hepatocyte necrosis in the liver, and villus atrophy in the intestine. Quantitative assessments further indicated that the co-exposure provoked the strongest oxidative stress response, with the highest increases observed during the acute phase—reaching up to 75% for SOD, 51% for CAT, and 52% for MDA relative to the control group (*P* < 0.05).

**Discussion:**

This study revealed the severe toxic effects of combined exposure to Cd and β-CP on zebrafish, underscoring the need for increased attention to the potential ecological risks of heavy metal and pesticide pollution in aquatic environments.

## 1 Introduction

Nowadays, Heavy metals are recognized as a primary concern in global environmental pollution (Liu et al., 2016; Luo et al., 2020). Among them, cadmium (Cd) is particularly notorious due to its high toxicity, persistence, and bioaccumulative potential, posing a significant threat to aquatic ecosystems (Chen et al., 2023b; Kiran et al., 2022). Heavy metals are primarily released into water bodies through industrial discharges, agricultural runoff, and urban sewage, posing substantial threats to ecological balance and human health. Specifically, Cd pollution in aquatic ecosystems has become a pressing environmental problem (Xiang et al., 2021; Parakaw et al., 2025). Cd as a common and persistent environmental pollutant, poses a significant threat to aquatic ecosystems due to its high toxicity and bioaccumulative potential. (Hussain et al., 2021; Xie et al., 2021; Huang et al., 2023). In freshwater bodies, the concentration of Cd ranges from 0.01 μg/L to 0.5 μg/L, For instance, it ranges from 1.12 to 4.47 μg/L in the Luan River basin in China, 8.0 μg/L in the East Lake area of Wuhan, and can reach up to 800 μg/L in heavily polluted sections of the Pearl River (Li et al., 2018; Yan and Xie, 2015).

β-Cypermethrin (β-CP), a widely used pyrethroid insecticide, is highly toxic to aquatic organisms and exhibits considerable environmental persistence (Farag et al., 2021; Petrovici et al., 2025). Following agricultural application, it can enter water bodies via runoff or rainfall, where its persistence leads to long-term adverse effects. While its toxicity to insects is well-documented (Petrovici et al., 2020; Zhao et al., 2020; Liu et al., 2023), studies on its toxicological impact, particularly in adult zebrafish, remain limited. There are also relatively few studies on the acute toxicity of β-CP to adult zebrafish. This study uses adult zebrafish as research subjects, examining behavioral changes in antioxidant enzyme activity and MDA levels in tissues and organs to investigate the acute and chronic toxicity of β-CP on zebrafish, providing a basis for the systematic assessment of β-CP toxicity in zebrafish.

Zebrafish (*Danio rerio*) are a well-established model in environmental toxicology and water quality studies (Langa et al., 2021; Liu et al., 2024). Their high sensitivity to pollutants via gill absorption and ingestion, coupled with their short life cycle and rapid maturation, makes them particularly suitable for chronic toxicity studies within a practical timeframe (Yin et al., 2018; Sharma et al., 2021; Zhao et al., 2024). Critically, their fundamental biochemical pathways, including the oxidative stress response and neurotransmitter systems, are highly conserved with those of higher vertebrates. This conservation makes findings on pollutant-induced oxidative damage and neurotoxicity in zebrafish highly relevant for understanding potential risks in other species.

Oxidative stress results from an imbalance between the production of reactive oxygen species (ROS) and the antioxidant defenses of an organism. This imbalance leads to oxidative damage of cellular components such as lipids, proteins, and DNA. Various environmental contaminants can induce oxidative stress in non-target organisms, thereby elevating ROS levels (Chen et al., 2023a; Forman and Zhang, 2021; Mittler et al., 2022; Sies et al., 2022). To counteract oxidative damage, organisms utilize antioxidant enzymes such as catalase (CAT) and superoxide dismutase (SOD); CAT facilitates the breakdown of hydrogen peroxide into water and oxygen, while SOD catalyzes the dismutation of superoxide radicals into oxygen and hydrogen peroxide. (Engelbrecht et al., 2023). Consequently, the enzymatic activities of CAT and SOD, which directly neutralize ROS, along with the concentration of malondialdehyde (MDA), a terminal product of lipid peroxidation, are extensively utilized as synergistic biomarkers to comprehensively assess oxidative stress levels, antioxidant defense capacity, and the extent of environmental pollution (Xu et al., 2023; Zamora et al., 2023).

In natural aquatic environments, pollutants rarely exist in isolation; rather, they occur as complex mixtures whose combined toxicity may differ substantially from that of individual compounds. This interaction necessitates the study of chemical mixtures to accurately assess ecological risk. Jijie et al. investigated the acute effects of Cd, nickel, and deltamethrin on zebrafish were studied (Jijie et al., 2020), which showed that when deltamethrin is mixed with Cd/Ni, the toxicity in certain behavioral indicators and oxidative stress in zebrafish was significantly reduced. Therefore, studying the toxicity effects of a single compound cannot accurately estimate their impact on aquatic ecosystems. Shen et al. (Shen et al., 2020)studied the effects of malathion (MAL), chlorpyrifos (CHL), and *lambda*-cyhalothrin (LCY) both individually and in combination on zebrafish embryos, analyzing oxidative stress and immune system impacts to provide valuable insights into the toxicity effects of mixed compounds on zebrafish. These findings highlight a critical gap in our understanding and underscore the necessity of investigating the combined effects of common pollutants such as Cd and β-CP. We therefore hypothesized that co-exposure to Cd and β-CP would induce synergistic oxidative stress and histopathological damage in zebrafish, exceeding the effects of individual exposures.

Based on the foregoing rationale, this study was designed to evaluate the individual and combined toxicity of Cd and β-CP in adult zebrafish through both acute and chronic exposure paradigms. Specific objectives included 1) determining the acute LC50 and observing behavioral responses; 2) quantifying changes in oxidative stress biomarkers (SOD, CAT, MDA) in gill tissue; 3) assessing Cd and β-CP bioaccumulation; and 4) examining associated histopathological alterations in the brain, liver, and intestine.

## 2 Materials and methods

### 2.1 Experimental animals

The zebrafish (AB strain) used in the experiment were obtained from the Institute of Model Animals (IMA), Wuhan University. The fish were 3–4 months old and consisted of a mixed-sex population (male and female). They were kept in a constant temperature recirculating water system at 28 °C, with a pH of 6.7–7.2, and the water quality was maintained within optimal ranges: dissolved oxygen (DO) > 7.0 mg/L, ammonia <0.05 mg/L, and hardness at 50–100 mg/L as CaCO_3_. The fish were maintained under a daily light cycle of 14 h light/10 h dark. The zebrafish were fed a commercial zebrafish diet three times a day, and the water was regularly cleaned to ensure a clean living environment. A 15-day acclimation period was provided to ensure full adaptation to the experimental conditions. To ensure experimental accuracy, the zebrafish were fasted for 24 h prior to the initiation of toxicity exposure. During this 24-h acclimation period in the experimental chambers, no mortality was observed. Furthermore, to avoid the confounding effects of waste accumulation from feeding, no food was provided throughout the entire acute exposure period (96-h).

### 2.2 Chemicals

Laboratory-grade Cd chloride (CdCl_2_·2.5H_2_O) was used as the stock solution (analytical grade), The β-cypermethrin (β-CP) emulsifiable concentrate (active ingredient: 10%) was obtained from Hubei Ruibaode Biochemical Co., Ltd. (China) (Product Batch No.: 20220103H424). The acute and chronic concentrations of Cd and β-CP solutions were prepared. The acute experiment was a concentration gradient experiment with Cd concentrations of 0 mg/L (CK), 4.0 mg/L, 8.0 mg/L, 16 mg/L, and 32 mg/L, and β-CP concentrations of 0 μg/L (CK), 2.0 μg/L, 4.0 μg/L, 6.0 μg/L, and 8.0 μg/L. The chronic mixture concentration (1/10 96 h-LC50) was Cd 1.78 mg/L and β-CP 0.82 μg/L.

### 2.3 Experimental method

The toxicity test was conducted in accordance with the principles of the OECD Test Guideline for Chemicals (Guideline 203: Acute Toxicity Test on Fish). The experimental design included a shared control group, eight groups subjected to acute exposure, and one group subjected to chronic exposure. Each treatment group consisted of four independent replicates, with each replicate containing 10 fish, resulting in a total of 40 fish per treatment condition.

In the acute experiments, each group of zebrafish was exposed to a 1 L glass jar. The number of zebrafish deaths was observed and recorded, and the dead fish were promptly removed. Zebrafish mortality was judged by the following criteria: cessation of gill cover movement, lack of response to external stimuli, loss of buoyancy, and sinking to the bottom. The mortality rate within 96 h was calculated, and the half-lethal concentration (LC50) and 95% confidence interval were determined using the probability unit method. Behavioral responses and mortality of zebrafish exposed to specific chemicals were observed and recorded over a specified period. Changes in SOD and CAT enzyme activities, MDA levels, and levels of toxic substances *in vivo* were studied to assess the toxic effects of chemicals on aquatic organisms at higher concentrations.

In the chronic experiment, a semi-static (renewal) protocol was adopted. The test solutions and control water were completely renewed every 48 h. To verify the stability of the exposure concentrations, water samples were collected from the test jars immediately after renewal (0 h) and immediately before the next renewal (48 h) for analytical verification. The measured concentrations of Cd and β-CP were maintained within ±15% of the nominal concentrations throughout the study period. This protocol ensured stable exposure concentrations, removed metabolic wastes, and maintained adequate dissolved oxygen levels throughout the 60-day study.

### 2.4 Histopathological analysis

During the experiment, strict preservation methods were used for the dead zebrafish samples to ensure sample quality and integrity. Immediately after death, the zebrafish were placed in a 10% neutral formaldehyde fixative. Detailed records were kept after fixation, including sample quantity, time of death, exposure conditions, and other relevant information. Euthanasia was performed on zebrafish that were still alive at the end of the experiment. The euthanasia procedure involved anesthetizing the zebrafish with MS-222 (tricaine methanesulfonate) at a concentration of 320 μg/mL until loss of opercular movement was observed, ensuring a humane and painless death.

### 2.5 Sample testing

#### 2.5.1 Biochemical analysis

Superoxide dismutase (SOD) activity was determined using a commercial WST-1 assay kit (Nanjing Jianjian Bioengineering Institute, China), following the manufacturer’s instructions. The principle of the assay is that WST-1 reacts with superoxide anions generated by the xanthine oxidase system to produce a water-soluble formazan dye. SOD inhibits this reaction by scavenging the superoxide anions. The inhibition rate was measured colorimetrically. SOD activity was then calculated based on the inhibition rate and normalized to the total protein concentration of the sample, with results expressed as units per milligram of protein (U/mg prot). Total protein concentration was determined using the BCA method. Catalase activity (U/mg) was determined in zebrafish according to Aebi’s method (Aebi et al., 1984). Zebrafish were dissected to remove their livers and homogenized in ice-cold 50 mM potassium phosphate buffer (1:8 w/v [pH 7.0]). Subsequently, the homogenates were centrifuged at 10, 600 g at 4 °C for 15 min, after which the supernatant was immediately taken and assayed for enzyme activity and protein concentration at room temperature (22 °C). Calculation of catalase activity was performed based on the rate of change of UV absorbance. One unit of CAT activity was defined as the amount of enzyme required to semi-degrade H_2_O_2_ in the sample after 100 s of enzyme action at 22 °C. To reflect the enzyme activity more accurately, we needed to normalize it by protein concentration. Ultimately, the results of the assay were expressed as units of CAT activity per milligram of total protein as U/mg total protein.

Malondialdehyde (MDA) is a byproduct of lipid oxidation, which occurs when organisms experience oxidative stress. During this process, certain fatty acids are oxidized and degrade into various compounds, including MDA. The extent of lipid oxidation can be assessed by measuring MDA levels. In this study, MDA content was quantified using a test kit from Nanjing Jianjian Bioengineering Institute. The method involves a chromogenic reaction where MDA reacts with thiobarbituric acid (TBA) under acidic conditions and elevated temperatures, forming a red MDA-TBA adduct. This adduct has a peak absorption at 530 nm, allowing for quantification through colorimetric analysis. Determination of Cadmium in Biological Tissues: Approximately 0.1 g samples of zebrafish liver, intestine, and brain tissues were collected and rapidly frozen in liquid nitrogen. The frozen tissues were mechanically homogenized into a fine powder using a mortar and pestle maintained under liquid nitrogen. The powdered samples were quantitatively transferred to digestion vessels, and 10 mL of concentrated nitric acid (HNO_3_) was added to each. Microwave-assisted acid digestion was performed under controlled temperature and pressure conditions to ensure complete dissolution of the organic matrix. After digestion, the samples were cooled, carefully diluted to a final volume of 50 mL with ultrapure water (18.2 MΩ cm), and then filtered through a 0.45 μm nylon membrane filter.

Cadmium concentrations were determined using inductively coupled plasma mass spectrometry (ICP-MS) operated in He collision mode to eliminate polyatomic interferences. Quantification was achieved using an external calibration curve method with rhodium (Rh) as an internal standard to correct for instrumental drift and matrix effects. The method was validated using certified reference material (CRM TORT-3) with measured cadmium values showing 95%–105% recovery of certified values.

Detection of β-CP in Fish: Approximately 0.1 g of zebrafish liver, intestinal, and brain tissue samples were collected and finely ground under liquid nitrogen to obtain a homogeneous powder. Ultrasonication-assisted extraction was performed on the powdered samples using 10 mL of acetonitrile for 20 min, followed by centrifugation at 4,000 rpm for 10 min to separate the supernatant. The supernatant underwent further purification via a solid-phase extraction (SPE) column, after which the eluate was concentrated to dryness and reconstituted in 1 mL of acetonitrile. Analysis of β-CP was conducted using liquid chromatography-tandem mass spectrometry (LC-MS) on a C18 reversed-phase column, employing a mobile phase gradient and an electrospray ionization source in positive ion mode for enhanced mass spectrometry sensitivity. Detection was performed in multiple reaction monitoring (MRM) mode to target specific ion pairs of β-CP, with the detection wavelength optimized at 230 nm. Quantitative assessment was achieved via the internal standard method, allowing precise calculation of β-CP concentrations in the samples. The key method validation parameters for the determination of Cd and β-CP are summarized in Table 1.

**TABLE 1:** Method validation parameters for Cd (by ICP-MS) and β-CP (by LC-MS/MS) analysis.

Parameter	Cadmium (Cd)	β-Cypermethrin (β-CP)
LOD	0.005 mg/kg	0.002 mg/kg
LOQ	0.015 mg/kg	0.005 mg/kg
Recovery ± RSD	95%–105%	92%–98%

#### 2.5.2 Histopathological analysis

Standard hematoxylin and eosin (H&E) staining was performed on paraffin-embedded tissue sections (brain, liver, and intestine) according to established protocols. The stained sections were examined under a light microscope (Ci-S, Nikon), and high-resolution digital images were captured using a slide scanner (NanoZoomer-SQ, Hamamatsu) for subsequent histopathological evaluation.

### 2.6 Data analysis

The data were statistically analyzed using SPSS Version 22.0 (IBM, Armonk, NY, United States) and the data were graphed using Origin 2020 (OriginLab Corp., Northampton, MA, United States). Each value represents the mean ± standard deviation (SD) of four independent biological replicates (n = 4), where each replicate consisted of a sample from a distinct, individual zebrafish. Levels of statistical significance are shown as *P < 0.05 and **P < 0.01.

For comparisons involving multiple groups, the normality of data distribution and homogeneity of variances were first verified using the Shapiro-Wilk test and Levene’s test, respectively. If both assumptions were satisfied, a one-way analysis of variance (ANOVA) was applied, followed by Tukey’s honestly significant difference (HSD) *post hoc* test for pairwise comparisons. If the data violated either the normality or homoscedasticity assumption, the non-parametric Kruskal–Wallis test was employed instead, followed by Dunn’s test for pairwise comparisons.

The LC50 values for Cd and β-CP were estimated using Probit regression analysis. To enhance the robustness of the results, 95% confidence intervals were calculated and reported for each LC50 value, indicating the precision and reliability of the toxicity estimates.

## 3 Results

### 3.1 LC50 of zebrafish under acute exposure

The median lethal concentration (LC50) is a fundamental toxicological parameter that quantifies the concentration of a toxicant required to cause 50% mortality in a test population under defined conditions. It serves as a critical indicator for comparing toxicity potency between chemicals, with lower LC50 values indicating higher toxicity. As shown in Table 2, the LC50 values of Cd for zebrafish were 25.36, 22.94, 20.36, and 17.83 mg/L, as shown in Table 3, the LC50 values of β-CP for zebrafish are 6.41,4.96,4.23, and 3.75 μg/L for 24 h, 48 h, 72 h, and 96 h, respectively.

**TABLE 2:** Determination of 96 h LC50 of zebrafish by Cd (*P* < 0.05).

Exposure time/h	Regression equation	LC_50_ (mg/L)	95% confidence interval
24	y = 0.849x −0.996 *R* ^2^ = 0.924	25.36	25.36 ± 1.19
48	y = 0.373x −0.532 *R* ^2^ = 0.946	22.94	22.94 ± 1.03
72	y = 0.213x - 0.635 *R* ^2^ = 0.969	20.36	20.36 ± 0.78
96	y = 0.134x + 0.564 *R* ^2^ = 0.991	17.83	17.83 ± 0.64

**TABLE 3:** Determination of 96 h LC50 of zebrafish by β-CP (*P* < 0.05).

Exposure time/h	Regression equation	LC50 (µg/L)	95% confidence interval
24	y = 0.421x −1.333 *R* ^2^ = 0.921	6.41	6.41 ± 0.77
48	y = 0.492x −0.970 *R* ^2^ = 0.965	4.96	4.96 ± 0.52
72	y = 0.514x - 0.391 *R* ^2^ = 0.956	4.23	4.23 ± 0.41
96	y = 0.481x + 0.792 *R* ^2^ = 0.977	3.75	3.75 ± 0.23

### 3.2 Enzyme activity assay

**FIGURE 1:**
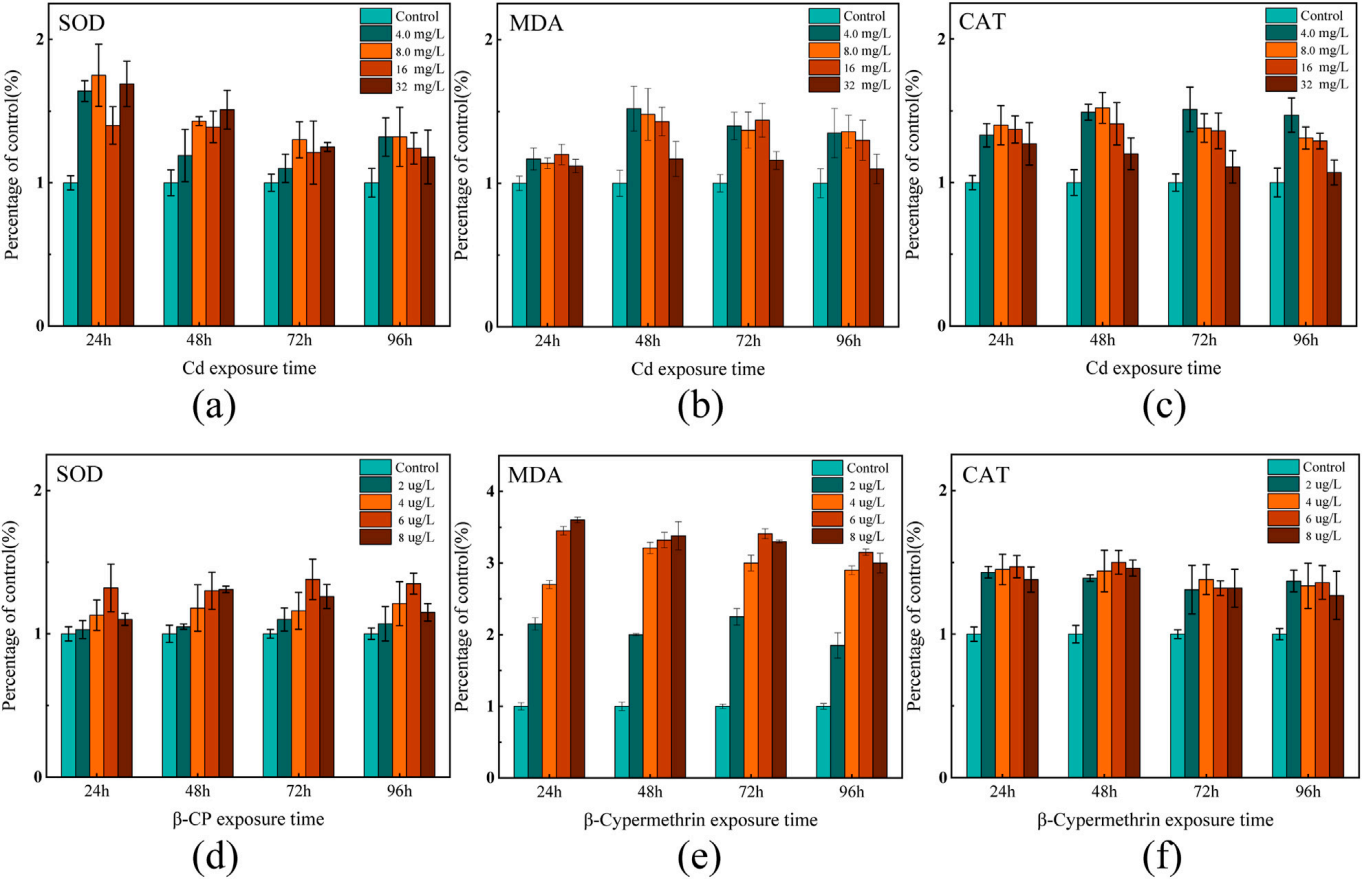
Effects of acute exposure on SOD and CAT enzyme activities and MDA levels in the gills of zebrafish **(a–c)** indicate changes in exposure to Cd; **(d–f)** indicate changes in exposure to β-CP.

This study examined the effects of acute exposure to Cd and β-CP on SOD and CAT activity and MDA levels in zebrafish gill tissue by measuring changes in SOD and CAT activity and MDA levels, as shown in Figure 1 (*P* < 0.05).

After 24 h of Cd exposure, SOD activity in the low-concentration Cd exposure groups (4.0 mg/L and 8.0 mg/L) increased by 64% and 75%, respectively, compared to the control group. However, as the exposure and concentration increased, SOD gradually decline. In the 96-h high-concentration exposure group (16 mg/L), SOD activity decreased by approximately 35% compared to the control group. MDA increased gradually with the Cd exposure duration and concentration. After 24 h of exposure, MDA levels slightly increased in all treatment groups compared to the control group, with no significant differences observed. After 48 h of exposure, MDA levels significantly increased in all treatment groups, particularly in the 4.0 mg/L and 8.0 mg/L groups, with increases of 52% and 48% respectively. After 72 and 96 h of exposure, MDA levels in all treatment groups were significantly higher than those in the control group, initially increasing and then slightly declining with higher Cd concentrations. This indicates an exacerbation of lipid peroxidation over time. Unlike the trends of SOD and MDA, CAT activity gradually decreased with increasing Cd exposure duration and concentration. In the 24-h low-concentration exposure group, CAT activity significantly increased in the 4.0 mg/L and 8.0 mg/L groups, rising by 33% and 41%, respectively, compared to the control group. After 48 h of exposure, CAT activity was higher in all treatment groups compared to the control group. By the 96-h high-concentration exposure, CAT activity significantly increased in the 4.0 mg/L and 8.0 mg/L groups, but significantly decreased in the 16 mg/L group, with CAT activity in the 32 mg/L group remaining roughly the same as the control group.

Zebrafish exhibited significant changes in enzyme activities after exposure to different concentrations of β-CP. SOD activity increased significantly at 24 h of exposure, with the 6.0 mg/L concentration group reaching a maximum at 24 h (132% of the control group), but gradually decreased in all treatment groups as the exposure time was extended to 48, 72, and 96 h. MDA content increased dramatically after 24 h of exposure, with the exposure groups (6 mg/L and 8 mg/L) reaching the maximum value at 24 h (345% and 360% of the control group), and then stabilized, while the MDA content of the 2.0 mg/L treatment group tended to stabilize at all exposure times. CAT activity significantly increased after 24 h of exposure, with CAT in all exposure groups remaining at a higher level. The CAT activity of the 6 mg/L group reached the maximum value at 48 h, which was about 51% higher than that of the control group, and then showed a decreasing trend. In the high-concentration groups (6.0 mg/L, 8.0 mg/L), CAT activity was significantly higher at 24 h of exposure, and then the trend leveled off. In all exposure groups, β-CP induced significant oxidative stress.

**FIGURE 2:**
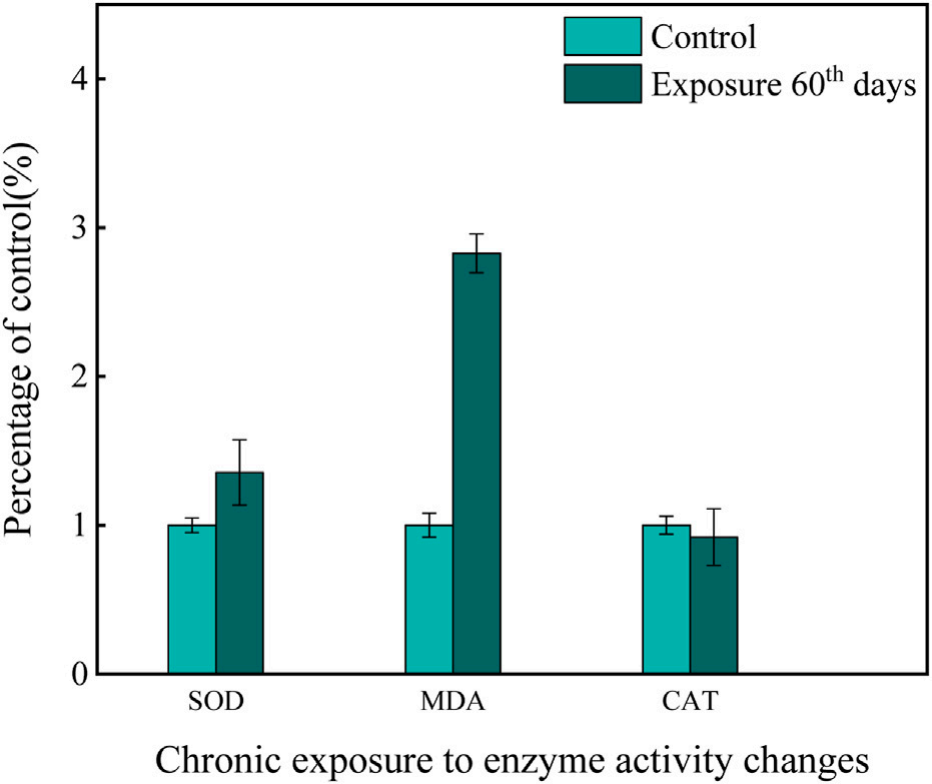
Effects of chronic exposure to a mixed solution on enzyme activity.

In addition to the acute experiment, a chronic study on enzyme activity in the gills of zebrafish exposed to a mixture of Cd and β-CP was conducted (Figure 2). After 60 days of chronic co-exposure, the levels of antioxidant enzymes and lipid peroxides in the gills were significantly altered. The activity of SOD increased from 379.71 ± 17.32 U/mg prot in the control group to 512.62 ± 24.36 U/mg prot in the exposed group, representing a 35% increase (*P* < 0.05). Similarly, the content of MDA rose markedly from 20.42 ± 0.93 nmol/mg prot to 57.61 ± 2.75 nmol/mg prot, corresponding to a 182% increase (*P* < 0.01). In contrast, CAT activity remained statistically unchanged, with values of 592.95 ± 28.48 U/mg prot and 563.31 ± 27.63 U/mg prot in the control and exposed groups, respectively (*P* > 0.05).

### 3.3 Heavy metal and pesticide residues in fish

After zebrafish were exposed to the chronic mixture for 60 days, the residues of Cd and β-CP in zebrafish were measured using liquid chromatography-tandem mass spectrometry (LC-MS), and the accumulation of the two compounds in their bodies was analyzed (Table 4). The results showed that the concentrations of Cd and β-CP in the fish were 1.21 ± 0.23 mg/kg and 0.48 ± 0.05 mg/kg, respectively. After 60 days of chronic exposure, the residues of Cd and β-CP in zebrafish were significantly higher than those in the control group, indicating that both compounds accumulated in the fish over time.

**TABLE 4:** Residual concentrations and bioconcentration factors (BCF) of Cd and β-CP in zebrafish under chronic exposure (n = 4).

Compound	Concentration (1/10 96 h-LC50)	Control group	Exposed group	BCF
Cd	1.78 mg/L	0.13 ± 0.017 mg/kg	1.21 ± 0.23 mg/kg*	0.68
β-CP	0.82 μg/L	0.03 ± 0.002 mg/kg	0.48 ± 0.05 mg/kg*	0.59

* Values are presented as mean ± SD. * indicates a significant difference from the control group (*P* < 0.05).*.

### 3.4 Effects of chronic exposure on the organization of zebrafish

**FIGURE 3:**
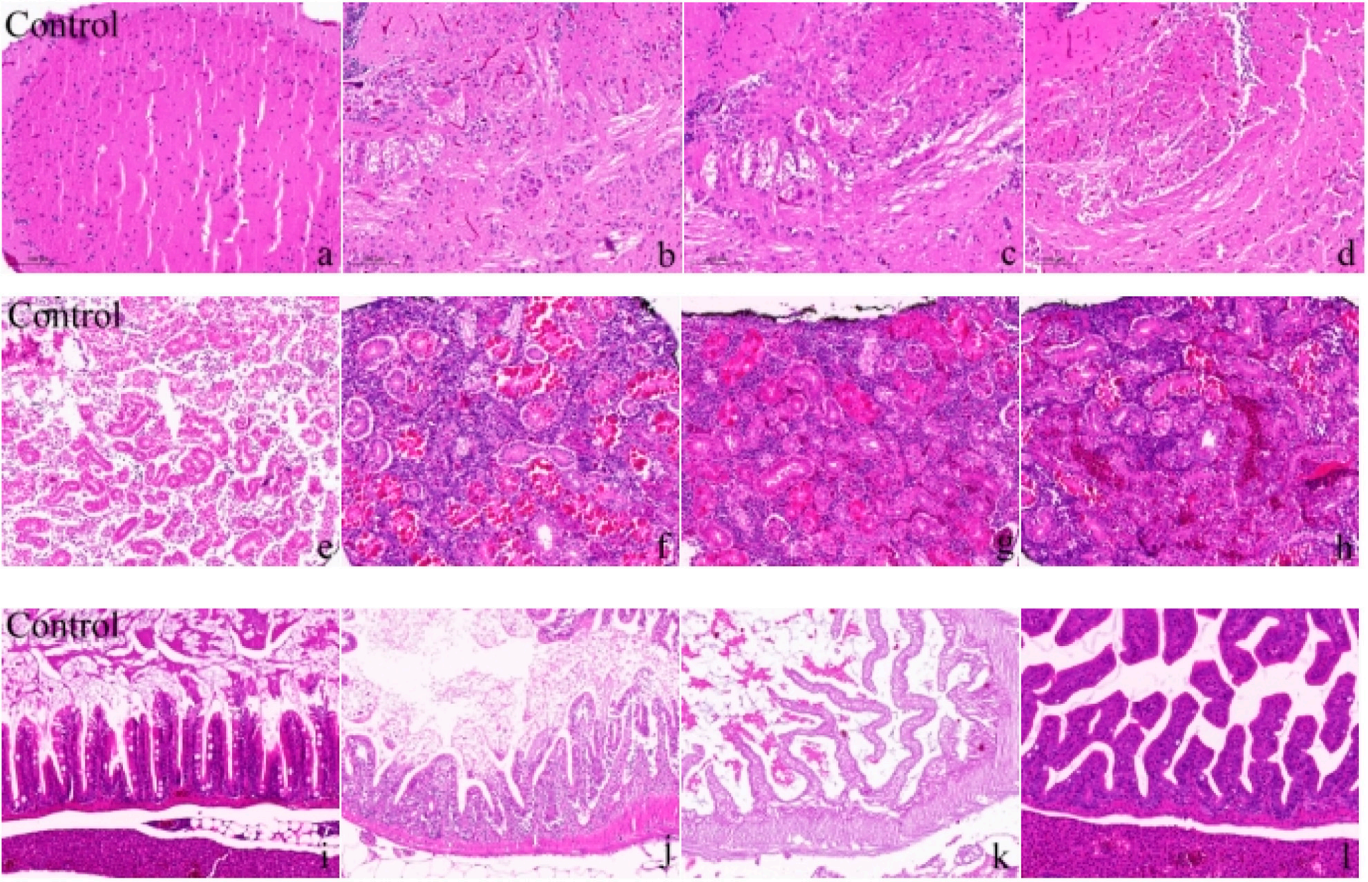
Morphological changes in the brain, liver, and intestine of zebrafish after 60 days of chronic exposure to the mixed solution **(a–d)** show the changes in zebrafish brain tissue; **(e–h)** show the changes in zebrafish liver tissue; **(i–l)** show the changes in zebrafish intestinal tissue). Scale bar in all panels represents 50 μm.

In the 60-day chronic exposure experiment, zebrafish exposed to a mixture of Cd (1.78 mg/L) and β-CP (0.82 μg/L) exhibited significant pathological changes. In the brain, liver, and intestines (Figure 3a). In the control group, the brain tissue structure was intact, with orderly cell arrangement and visible nuclei. In contrast, the exposed groups (Figures 3b-d) showed severe structural alterations, including disorganized cellular architecture, cytoplasmic vacuolization, nuclear fragmentation (karyorrhexis), as well as overall tissue loosening and expanded intercellular spaces. In the liver, the control group (Figure 3e) displayed normal hepatic architecture with tightly packed hepatocytes. The exposed groups (Figures 3f-h), however, revealed substantial damage characterized by loss of normal hepatocyte cord arrangement, cytoplasmic vacuolization, nuclear pylknosis (condensation) and fragmentation, and loosened parenchymal structure. Furthermore, areas of cell degeneration and disruption of the typical hepatic lobule pattern were evident.

After 60 days of chronic exposure to the mixed solution. Figures 3i-l illustrate the changes in zebrafish intestinal tissue, with Figure 3i representing the control group and Figures 3j–l representing the exposure groups. The control group exhibited intact intestinal tissue structure, with neatly arranged villi and high tissue density. Figures 3j–l in the exposure groups displayed significant tissue structural damage, including villus atrophy, disordered arrangement, loose tissue, and cellular degeneration. Additionally, irregular arrangement of mucosal layer cells, cell necrosis in some areas, and lax intestinal wall structure were noted. These changes indicate that long-term exposure to Cd and β-CP mixture induced significant toxic effects on zebrafish intestinal tissue, resulting in severe structural damage.

## 4 Discussion

During the acute exposure experiment, no deaths were observed in the control group, whereas all treatment groups showed mortality. Observations from the experiments on zebrafish revealed that varying concentrations of Cd and β-CP significantly influenced the poisoning symptoms. When zebrafish were exposed to high concentrations of β-CP, their reactions were notably evident. The primary symptoms included body curling, convulsions, accelerated opercular movements, and significantly reduced swimming ability. This acute neurotoxicity often culminated in death within the first 2–4 h of exposure in the highest concentration groups. In contrast, when zebrafish were exposed to high concentrations of Cd, poisoning symptoms manifested later and were relatively mild. This is consistent with the findings of Liao and Wang et al. (Liao et al., 2021; Wang et al., 2022), but after a period of exposure, some zebrafish still showed similar symptoms such as body curling and reduced swimming ability, but the overall symptoms were relatively mild.

This study reveals that β-CP exhibits exceptionally high acute toxicity in zebrafish, as indicated by pronounced behavioral responses following exposure. In contrast, zebrafish exposed to high concentrations of Cd displayed delayed and relatively milder symptoms. Nevertheless, significant effects were still observed (*P* < 0.05), suggesting that the cumulative impact of Cd could pose substantial long-term risks. These results are consistent with findings reported in previous studies. (Kim et al., 2011; Liu et al., 2021).

Results demonstrate that co-exposure to Cd and β-CP induced more severe oxidative stress and histopathological damage than individual exposures, indicating a synergistic toxic interaction. The oxidative stress biomarkers revealed a critical pattern: an initial compensatory upregulation of SOD and CAT activities was followed by their subsequent suppression, coupled with a marked elevation of MDA. This biphasic response is indicative of “oxidative exhaustion,” where the antioxidant defense system is overwhelmed under sustained chemical stress. The significant bioaccumulation of both compounds likely contributed to this process, ultimately leading to the observed severe damage in the brain, liver, and intestine. After 60 days of chronic mixed exposure, the activities of the antioxidant enzyme SOD and the lipid peroxidation product MDA in the gills of zebrafish increased significantly by 35% and 182%, respectively, indicating that zebrafish experienced strong oxidative stress and severe lipid peroxidation under chronic pollution conditions. However, CAT activity did not change significantly and slightly decreased, which may be related to the complex regulation of the antioxidant defense mechanism. These changes reveal the cumulative effects and potential hazards of chronic pollution on oxidative stress and the antioxidant defense system in zebrafish. This result is similar to several studies (Hu et al., 2022). The chronic exposure experiment confirmed the toxic effects observed under acute exposure and further demonstrated the comprehensive impacts of long-term exposure.

The distinct patterns of oxidative stress induced by Cd and β-CP likely stem from their fundamentally different mechanisms of action. Cadmium primarily exerts its toxicity by binding to sulfhydryl (-SH) groups in proteins (e.g., antioxidant enzymes such as SOD and CAT), leading to their inactivation and disruption of cellular redox homeostasis (Rahaman et al., 2021). This direct inhibition can explain the observed decline in enzyme activity upon prolonged exposure. In contrast, β-cypermethrin, as a pyrethroid insecticide, is known to disrupt mitochondrial function, leading to enhanced generation of reactive oxygen species (ROS) at the electron transport chain (Sun et al., 2022). This primary surge in ROS production imposes a greater initial burden on the antioxidant system, accounting for the more dramatic increase in MDA and the eventual exhaustion and suppression of SOD and CAT activities, particularly under high-concentration, long-term exposure scenarios.

In zebrafish, the residual concentrations of Cd and β-CP gradually increase with the extension of exposure time. These toxic substances accumulate in the vital organs of zebrafish, leading to significant biotoxic effects. This toxic impact is not limited to individual zebrafish but adversely affects their physiological functions, reproductive capacity, and survival rate. Other studies have demonstrated that these substances can transfer through the food chain (Tang et al., 2021; Mamun et al., 2023), affecting higher trophic-level predators such as fish, birds, and mammals.

Based on the light microscopy analysis of H&E-stained sections of zebrafish brain, liver, and intestine, it is evident that chronic exposure to the mixed solution for 60 days caused significant structural damage to several vital organs. In the photomicrographs of brain tissue, the control group (Figure a) shows an intact brain structure with tightly and orderly arranged cells. However, the exposed groups (Figures b, c, d) show significant tissue damage, including neuronal degeneration, disorganized structures, and increased intercellular spaces, indicating that long-term exposure to Cd and β-CP caused severe neurotoxic effects on the zebrafish central nervous system. The photomicrographs of liver tissue also show significant toxic effects. In the control group (Figure e), the liver cells have a clear structure, orderly arrangement, and normal nuclear staining. The exposed groups (Figures f, g, h) show swollen liver cells, disordered arrangement, and accompanying cell necrosis and tissue fibrosis. These pathological changes indicate severe damage to liver function and structure due to mixed solution exposure. The scanning electron micrographs of intestine tissue (Figure 3i for the control group, Figures j, k, l for the exposed groups) further confirm the toxic effects. The control group shows neatly arranged and intact intestinal villi, while the exposed groups show atrophied, disordered, loose tissue and cell degeneration, indicating that the mixed solution exposure led to the destruction of intestinal absorption function and severe tissue damage. Chronic mixed exposure caused multiple toxic effects on the brain, liver, and intestine tissues of zebrafish (Wang et al., 2021; Jheng et al., 2021).

These significant changes in tissue structure indicate profound damage at the tissue and cellular levels and suggest a severe threat to the overall physiological functions of zebrafish. Notably, the chronic exposure concentration of Cd (1.78 mg/L) used in this study is several orders of magnitude higher than the typical levels found in freshwater bodies (e.g., 0.01–0.5 μg/L), while the β-CP concentration (0.82 μg/L) falls within the range that could occur in agricultural runoff. This comparison underscores that even though the Cd concentration was elevated for experimental purposes, the combined exposure scenario, particularly with environmentally relevant levels of β-CP, can induce significant harm, highlighting a tangible ecological risk.

## 5 conclusion

This study comprehensively analyzed the toxic effects of acute and chronic exposure to Cd and β-CP on zebrafish, revealing multiple impacts of these chemical compounds on the physiological systems of zebrafish. The acute exposure experiments showed that β-CP has stronger acute toxicity, causing zebrafish to quickly exhibit obvious poisoning symptoms, whereas Cd’s acute toxicity is relatively mild but still induces oxidative stress responses, suggesting that Cd accumulation poses a considerable long-term risk. Chronic co-exposure to cadmium (Cd) and β-cypermethrin (β-CP) induced synergistic toxicity in zebrafish, as evidenced by exacerbated oxidative stress and histopathological damage. The co-exposure group exhibited a greater-than-additive effect, marked by a pronounced 182% increase in malondialdehyde (MDA) and severe structural alterations in the brain, liver, and intestine. These results demonstrate that the combination of Cd and β-CP poses a heightened ecological risk due to their synergistic interaction.

Research findings indicate that combined exposure to Cd and β-CP causes synergistic oxidative and histopathological damage in zebrafish, underscoring the necessity for mixture-based ecotoxicological risk assessments. This not only deepens our understanding of the mechanisms of fish growth and development but also provides a more scientific and comprehensive theoretical basis for environmental risk assessment and the formulation of ecological protection strategies.

## Data Availability

The raw data supporting the conclusions of this article will be made available by the authors, without undue reservation.
